# Does the number of free nicotine patches given to smokers calling a quitline influence quit rates: results from a quasi-experimental study

**DOI:** 10.1186/1471-2458-10-181

**Published:** 2010-04-07

**Authors:** K Michael Cummings, Brian V Fix, Paula Celestino, Andrew Hyland, Martin Mahoney, Deborah J Ossip, Ursula Bauer

**Affiliations:** 1 Department of Health Behavior, Roswell Park Cancer Institute, Buffalo, NY, USA; 2 Department of Community and Preventive Medicine, University of Rochester, Rochester, NY, USA; 3 Division of Chronic Disease Prevention and Adult Health, New York State Department of Health, New York, NY, USA

## Abstract

**Background:**

The offer of free nicotine replacement therapy (NRT) can be a cost-effective marketing strategy to induce smokers to call a telephone quitline for quitting assistance. However, the most cost-effective supply of free NRT to provide to smokers who call a quitline remains unknown. This study tests the hypothesis that smokers who call a telephone quitline and are given more free nicotine patches would report higher quit rates upon follow-up 12 months later.

**Methods:**

A quasi-experimental design was used to assess nicotine patch usage patterns and quit rates among five groups of smokers who called the New York State Smokers' Quitline (NYSSQL) between April 2003 and May 2006 and were mailed 2-, 4-, 6- or 8-week supplies of free nicotine patches. The study population included 2,442 adult (aged 18 years or older) current daily smokers of 10 or more cigarettes per day, who were willing to make a quit attempt, and reported no contraindications for using the nicotine patch. Outcome variables assessed included the percentage of smokers who reported that they had not smoked for at least 7-days at the time of a 12 months telephone follow-up survey, sustained quitting, delayed quitting and nicotine patch use.

**Results:**

Quit rates measured at 12 months were higher for smokers in the groups who received either 2, 6, or 8 weeks of free patches. The lowest quit rate was observed among the group of Medicaid/uninsured smokers who were eligible to receive up to six weeks of free patches. The quit rate for the 4-week supply group did not differ significantly from the 6-week or 8-week groups. These patterns remained similar in an intent-to-treat analysis of 12-month quit rates and in an analysis of sustained quitting.

**Conclusion:**

No clear cut dose response relationship was observed between the number of free nicotine patches sent to smokers and smoking outcomes. Baseline diferences in the characteristics of the groups compared could account for the null findings, and a more definitive randomized trial is warranted.

## Background

Research has demonstrated that the offer of free nicotine replacement therapy (NRT) can be a cost-effective marketing strategy to induce large numbers of smokers to call a telephone quitline for quitting assistance [1-3]. Our research has also shown that quit rates are increased in those who received NRT in addition to telephone counseling support compared to those who received counseling support alone [4]. However, the most cost-effective supply of free NRT to provide to smokers who call a quitline remains unknown.

The labeling for nicotine medications recommends that smokers use the medication for at least eight to 12 weeks. This recommendation is based on the observation that most of those trying to stop smoking relapse back to smoking within the first three months of quitting [5,6]. Clinical trials evaluating the efficacy of NRT have typically offered smokers a minimum of eight to 12 weeks of treatment [7]. While studies have tended to find a positive correlation between the duration of NRT use and cessation, it is not clear from these studies whether giving more medication to smokers actually increases their odds of quitting. For example, a reasonable alternative explanation for the observed positive association between the use of NRT and quit rates is that those who are no longer smoking remain compliant with using the medication while those who relapse back to smoking discontinue use of the medication [8-12]. Only two randomized trials have examined the effects of varying the duration of NRT, and neither found any benefit from extending treatment from 12 weeks to 18 or 26 weeks [10,11]. However, neither of these studies addressed the value of giving smokers different amounts of free medications at the outset of their quit attempt.

NRT helps people to stop smoking by lessening nicotine withdrawal symptoms after quitting which for many smokers last about two weeks [13-17]. The relatively short duration of nicotine withdrawal symptoms is consistent with our previously reported observation of similar quit rates among smokers given a 1-, 2-, or 6-week supplies of free nicotine patches [2].

The data presented in this paper extend our earlier studies comparing patch usage patterns and quit rates among smokers given different amounts of free patches [2,4]. Subjects in this study included adult smokers who contacted the New York State Smokers' Quitline (NYSSQL) and received a 2-, 4-, 6-, or 8-week supplies of nicotine patches along with a stop smoking guide and brief telephone counseling. The data from this study allow us to test the hypothesis that smokers given more nicotine patches would report higher quit rates upon follow-up 12 months later.

## Methods

A quasi-experimental design was used to compare five groups of smokers (n = 2,442) who received free nicotine patches mailed to them by the NYSSQL between April 2003 and May 2006. The groups were similar in that all subjects had to meet the same core criteria in order to be eligible to get the free nicotine patches made available through the NYSSQL. These core criteria required participants to be 18 years of age or older, current daily smokers of 10 or more cigarettes per day, willing to make a quit attempt in the next fourteen days (seven days for the 6 week program), receive a follow-up phone call to determine their smoking status, and report no contraindications for using the nicotine patch. In addition to the free nicotine patches, participants were mailed an instruction sheet on how to use the medications and a copy of the NYSSQL's Break Loose stop smoking guide. As part of routine service, the NYSSQL attempted to call all smokers who received the free nicotine patches within two to four weeks of shipment of the NRT. The purpose of the callback was to make sure callers received the nicotine patches and understood the information packet, and to provide counseling support to assist their quit attempt. Medicaid and uninsured smokers (Group 3 described below) received up to four additional proactive counseling calls.

The availability of the free NRT was promoted through various media strategies, including press releases, TV, radio, direct mail, word of mouth, and print advertisments throughout New York State. Different promotional strategies were used at different times and overlapped among the groups.

### Five Groups of Smokers

While all subjects were similar with regards to the core eligibility criteria for getting the free patches, the five groups of subjects did differ in terms of the time frame when they were recruited into the study, the amount of free nicotine patches mailed to them, and health insurance status. Table 1 gives a brief description of each of the five groups of smokers compared in this study, and each of these groups is briefly described below.

**Table 1:** Description of the five groups of smokers given free nicotine patches

Amount of free nicotine patches sent to smokers	Group 1 2-week supply	Group 2 4-week supply	Group 3 6-week supply contingent upon first 2-weeks of use	Group 4 6-week supply	Group 5 8-week supply
Number of Participants	19,852	38,667	19,859	35,334	2,000
Survey Sample Size	490	588	731	1,386	707
Program Dates	11/2004 - 8/2005	9/2005 - 5/2006	11/2004 - 5/2006	4/2003 - 5/2003	4/2004 - 6/2004
Mean duration of follow-up for the initial follow-up survey (months)	3.14	3.12	3.15	5.51	7.24
Response rate initial follow-up survey*	69.0%	69.0%	45.2%	63.7%	68.7%
					
Number of eligible subjects who completed the initial follow-up survey	338	406	332	880	486
Mean duration of follow-up for the 12-month follow-up survey (months)	12.85	12.08	12.03	14.00	12.50
Response rate 12-month follow-up survey**	68.6%	56.9%	50.0%	65.6%	73.3%
					
Number of eligible subjects who completed both surveys	232	231	166	577	356
Overall response rate 12-month follow-up survey***	47.3%	39.3%	22.7%	41.6%	50.3%

*Initial follow-up response rate defined as the number of completed interviews among those considered eligible for follow-up (defined as those with working phone numbers).

**12-month response rate defined as the number of completed interviews among those who completed the initial follow-up survey

***Overall response rate defined as the number of completed interviews among those initially eligible for follow-up

The first group of subjects consists of 19,852 privately insured smokers across New York State who called the NYSSQL between November 2004 and August 2005, when the NYSSQL began routinely providing free 2-week "starter kits" of either 21 mg or 14 mg nicotine patches.

The second group of subjects consists of 38,667 privately insured smokers across New New York State who called the NYSSQL between September 2005 through May 2006, when a special promotion provided a starter kit containing a 4-week supply of either 21 mg or 14 mg nicotine patches.

The third group of subjects consists of 19,589 Medicaid or uninsured smokers across New York State who called the NYSSQL between November 2004 and May 2006. Medicaid and uninsured smokers were eligible to receive up to 6-weeks of free nicotine patches (i.e., 4 weeks of 21 mg patches plus 2 weeks of 14 mg patches, or 4 weeks of 14 mg patches plus 2 weeks of 7 mg patches) contingent upon their agreement to receive up to four proactive counseling calls from the NYSSQL and reporting of progress towards quitting (i.e., used the patches previously sent to them, motivated to continue with quit plan) within two weeks of receiving their 2-week or 4-week starter kit of free patches.

The fourth group of smokers to receive free nicotine patches from the NYSSQL resided in New York City and was offered a 6-week supply between April 2 and May 14, 2003. The free patch give away program was sponsored by the New York City Department of Health and Mental Hygiene in collaboration with the NYSSQL and coincided with implementation of New York City's smoke-free workplace law. In a single mailing, 35,334 eligible smokers, regardless of health insurance status, were sent a 4-week supply of 21 mg nicotine patches and a 2-week supply of 14 mg patches.

The fifth group consisted of 2,000 smokers identified between April and June 2004, who had no health insurance and were mailed a single package containing a total of 8 weeks of nicotine patches as follows: a 4 week supply of 21 mg patches, a 2 week supply of 14 mg patches, and a 2 week supply of 7 mg patches.

### Follow-up of enrolled smokers

To evaluate patch usage patterns, smoking behavior, and satisfaction with service outcomes, a standardized brief telephone follow-up interview with a random sample of callers was conducted a two points following subject enrollment. Initially, subjects were contacted by telephone between three and seven months (on average) after contacting the quitline. Those who completed the initial telephone follow-up interview were recontacted again at 12 months after enrollment to reassess smoking status. The telephone survey protocol was developed at Roswell Park Cancer Institute and approved by its Institutional Review Board.

The main outcome variable examined in this paper is the percentage of smokers who reported that they had quit smoking at the time of the 12 month follow-up interview. Subjects who reported that they had not smoked in the 7 days prior to the follow-up interview were defined as having quit smoking.

The 7-day point prevalence quit rate at 12 months follow-up was computed in two ways. First, we estimated the quit rate at the time of the 12 month follow-up interview for each group based on the achieved sample, under the assumption that those lost to follow-up were missing at random. Second, we estimated the quit rate for each group based on an intent-to-treat basis assuming that those lost to follow-up had all returned to smoking.

Also, since we assessed smoking status at two different times after enrollment in the program we were able to compute three additional smoking outcome measures, one reflecting sustained quitting, another reflecting relapse back to smoking, and the third measuring delayed quitting. Sustained quitting was defined as the percentage of subjects in each group who reported not smoking at both the initial follow-up interview and again at the time of the 12 month follow-up interview. Relapsed back to smoking was defined as the percentage of subjects who at the time of the initial follow-up interview were not smoking, but who reported that they were smoking again when contacted at the time of the 12 month follow-up interview. Delayed quitting was defined as the percentage of subjects who were smoking at the time of the initial follow-up interview who were not smoking at the time of the 12 month follow-up interview.

Nicotine patch usage was assessed by asking subjects at the time of their initial follow-up survey to report how many of the patches sent to them in the mail they had used and if they had purchased additional NRT beyond those sent for free in the mail. For analysis purposes we categorized subjects by the number of free patches used: zero, 1-7, 8-14, 15-28, 29-42, >43 patches used.

Table 1 shows the number of participants, the survey sample size, and response rates for each of the five groups. The response rates varied significantly between the groups ranging from 23% to 50%. Compared to responders, non-responders in each group were also signicantly more likely to be younger age (i.e., 18-35 years compared to >35 years) and non-white (data not shown). Non-responders to the 12 month follow-up interviewer were also somewhat more likely to report smoking at the time of the initial follow-up interview compared to responders.

### Statistical Analysis

All data analyses were conducted using SPSS 14.0. Descriptive statistics from the initial follow-up telephone surveys were used to assess patterns of nicotine patch use and to describe smoking outcomes for each group. To compare differences between groups in their quit rate measured at 12 months we used analysis of covariance to compare the mean differences for each of the five outcome variables between each group adjusted for the following potential confounders: age, gender, race (categorized as: White-Non-Hispanic, Black-Non-Hispanic, Hispanic, and other), education (dichotomized as: level high school graduate or less, more years of education beyond high school), and number of cigarettes smoke per day (CPD) at time of enrollment. Since this was an exploratory study with multiple comparisons (10 for each outcome measure) made between groups, we have adjusted p-values using the Bonferroni correction to try to avoid making any spurious conclusions about group differences [18].

### Characteristics of the Five Groups

Table 2 shows that the five study groups were similar in terms of gender and number of cigarettes smoked per day. The groups also differed in expected ways with respect to age, race/ethnicity, education, and health insurance status. Specifically, participants in Group 3 (Medicaid/uninsured group) and Group 5 (uninsured only group) were younger and less educated compared to the other groups. Groups 3, 4 and 5 also included more diverse smokers based on race and ethnicity compared to Groups 1 and 2. By definition, Groups 1 and 2 included only privately insured callers while Group 5 included only uninsured callers. Group 3 consisted of 41% Medicaid enrolled subjects and 59% uninsured. Group 4 participants (NYC smokers who received 6-weeks of NRT) included 69% privately insured, 18% Medicaid insured, 10% uninsured and 3% other or unknown insurance status.

**Table 2:** Characteristics of the five groups of smokers given free nicotine patches

Amount of free nicotine patches sent to smokers	Group 1 2-week supply	Group 2 4-week supply	Group 3 6-week supply contingent upon first 2-weeks of use	Group 4 6-week supply	Group 5 8-week supply
CPD at Baseline					
10-19 CPD	31.1%	29.0%	28.4%	23.5%	30.3%
20-30 CPD	40.8%	44.6%	40.5%	43.9%	50.4%
31 + CPD	28.1%	26.5%	31.1%	32.6%	19.2%
Gender					
Male:	45.0%	42.1%	48.5%	43.4%	47.9%
Female:	55.0%	57.9%	51.5%	56.6%	52.1%
Age†					
18-34:	22.5%	23.2%	37.7%	21.8%	31.3%
35-54:	52.1%	57.4%	53.3%	57.8%	52.9%
55+:	25.4%	19.5%	9.0%	20.4%	15.8%
Race/ethnicity†					
White non-Hispanic	72.2%	81.0%	64.8%	52.1%	65.4%
Black non-Hispanic	14.2%	9.9%	17.2%	25.9%	18.6%
Hispanic	7.4%	6.7%	13.3%	14.9%	8.9%
Other	6.2%	2.5%	4.8%	7.2%	7.2%
Education†					
High School or Less	46.7%	42.7%	54.9%	43.4%	53.2%
Greater than High School	53.3%	57.3%	45.1%	56.6%	46.8%
Health Insurance					
Private	100.0%	100.0%	00.0	69.3%	0.0%
Medicaid	0.0%	0.0%	40.6	18.3%	0.0%
Uninsured	0.0%	0.0%	59.4	9.6%	100.0%
Other/unknown	0.0%	0.0%	00.0	2.8%	0.0%

† Age, Race/ethnicity, Education - Chi-square test: p-value < 0.01.

## Results and Discussion

### NRT Usage Patterns

Table 3 describes the nicotine patch usage patterns across the five groups. The number of patches used did differ significantly between groups with a chi-square test resulting in a p-value of < 0.01. Most subjects reported using at least some of the patches sent to them in the mail, with generally similar usage patterns observed for Groups 1 to 3 and for Groups 4 and 5. The number of patches reportedly used was related to the amount of free patches sent to subjects, with a significantly greater number of patches used by those receiving the 6- and 8-week supplies relative to those who only received a 2- or 4-week supply of free patches, with the exception of those who received the 6-week supply of free patches contingent upon use of the initial supply of patches sent to them. Members of Group 3 (6 weeks supply contingent upon first two weeks of use) were about as likely as those who received 4-weeks of free patches to report using more than 14 patches and substantially less likely to report using more than 14 patches compared to in Groups 4 and 5. The number of program participants who reported obtaining an additional supply of nicotine patches, beyond the patches furnished by the NYSSQL, was 11%. However, participants enrolled in the 2-week, 4-week and 6 week contingent programs (26.0%, 17.0%, 17.8% respectively) were more likely to have acquired additional patches compared to callers who participated in the 6-week and 8-week programs (2.8% and 3.1%, respectively).

**Table 3:** Description of Nicotine Patch Usage

Number of free nicotine patches provided	Group 1 2-week supply	Group 2 4-week supply	Group 3 6-week supply contingent upon first 2-weeks of use	Group 4 6-week supply	Group 5 8-week supply
Number completed surveys	338	406	332	884	486
% Received free patches	97.9%	94.1%	98.2%	98.9%	98.6%
% Used any of the free patches	84.3%	82.3%	88.6%	88.9%	87.8%
Number of patches used†					
0 patches	14.3%	15.2%	11.7%	11.4%	2.6%
1-7 patches	22.9%	24.9%	19.4%	16.9%	17.6%
8-14 patches	61.6%	43.0%	52.8%	10.6%	9.5%
15-28 patches	0.9%	12.6%	10.8%	22.0%	16.2%
29-42 patches	0.3%	3.4%	3.7%	39.2%	16.9%
43 + patches	0.0%	0.8%	1.5%	11.4%	37.1%
% Purchased additional NRT†	26.0%	17.0%	17.8%	2.8%	3.1%

† Chi-square test: p-value < 0.01.

### Smoking Outcomes

The smoking status outcomes for each of the five groups are summarized in Table 4. Quit rates measured at 12 months were higher for smokers in the groups who received either 2 weeks of free patches (Group 1) or the full 6 weeks (Group 4) or 8 weeks (Groups 5) of free patches. The lowest quit rate was observed among the group of Medicaid/uninsured smokers who were eligible to receive up to six weeks of free patches, contingent on their quitting efforts made within the first two weeks of calling the quitline. The quit rate for Group 2 did not differ significantly from Groups 4 or 5.

**Table 4:** Smoking Outcomes by the Amount of Free Nicotine Patches Sent to Smokers

Number of free nicotine patches provided	Group 1 2-week supply	Group 2 4-week supply	Group 3 6-week supply contingent upon first 2-weeks of use	Group 4 6-week supply	Group 5 8-week supply
12-month quit rate*	31.0%	21.0%	15.5%	37.8%	31.1%
12-month intent-to-treat quit rate**	19.1%	12.9%	7.0%	23.1%	22.6%
Sustained quitters*	24.6%	20.3%	11.7%	27.3%	19.9%
Relapsed smokers*	18.9%	14.9%	16.4%	11.9%	10.2%
Delayed quitters*	6.4%	0.7%	3.8%	10.5%	11.3%

* adjusting for demographic characteristics (age, gender, and race), education level, and number of cigarettes smoked per day at the time of enrollment.

**non-responders are counted as smokers at the time of the 12-month follow-up survey.

Table 5 shows the mean differences between groups on all smoking outcome measures. The asterisk signifies that the differences between groups that are statistically significant at the p < 0.05 level. What is evident from the pattern of differences displayed in the table is that there is no consistent dose response relationship between the number of free patches given to smokers and smoking status outcomes. For example, only three group differences reached statistical significant at the p < 0.05 level on our measure of 7-day nonsmoking prevalence at 12 months: group 2 (received 4 weeks of free patches) had a lower quit rate relative to group 4 (New York City group that received 6-week supply of patches); group 3 (eligible for 6 weeks of free patches contingent upon first 2 weeks) had a lower quit rate compared with groups 4 and 5 (New York City group that received 6 weeks and uninsured group that received 8 weeks of free patches). Notably, for the two groups that are most similar in sampling and demographics (Groups 1 and 2), no differences were found in quit rates for 2- vs. 4-week supplies. Comparing the 2-week group (Group 1) to the next most similar group (Group 4, received 6-week supply), again no differences were found in quit rates. For the two Medicaid and/or uninsured groups (Groups 3 and 5), quit rates were significantly higher in the 8-week supply group (Group 5), though not different from the 2-week insured group (Group 1). The pattern of differences observed between groups remains essentially the same for the other outcome measures assessed.

**Table 5:** Mean differences on smoking outcome measures between groups

		Mean Difference on Smoking Outcome Measures				
		
Comparisons		12-month quit rate	12-month intent-to-treat quit rate	Sustained quitters	Relapsed smokers	Delayed quitters
**Group 1** 2-week supply	**Group 2** 4-week supply	.05	.06	.01	.00	.03
**Group 1** 2-week supply	**Group 3** 6-week supply contingent upon first 2-weeks of use	.07	.08*	.05	-.01	.02
**Group 1** 2-week supply	**Group 4** 6-week supply	-.08	-.04	-.04	.08*	-.04
**Group 1** 2-week supply	**Group 5** 8-week supply	-.05	-.05	-.03	.09*	-.02
**Group 2** 4-week supply	**Group 3** 6-week supply contingent upon first 2-weeks of use	.03	.03	.04	-.02	-.01
**Group 2** 4-week supply	**Group 4** 6-week supply	-.12*	-.10*	-.05	.07	-.07*
**Group 2** 4-week supply	**Group 5** 8-week supply	-.10	-.11*	-.04	.09*	-.06
**Group 3** 6-week supply contingent upon first 2-weeks of use	**Group 4** 6-week supply	-.15*	-.13*	-.09	.09*	-.06
**Group 3** 6-week supply contingent upon first 2-weeks of use	**Group 5** 8-week supply	-.13*	-.14*	-.08	.10*	-.04
**Group 4** 6-week supply	**Group 5** 8-week supply	.02	-.01	.01	.02	.01

Note: Group 1 = 2 weeks of patches; Group 2 = 4 weeks of patches; Group 3 = 6 weeks of patches contingent upon progress toward quitting measured at 2 weeks; Group 4 = 6-weeks of patches; Group 5 = 8 weeks of patches

* indicates significant difference in means between group compared is different at p < 0.05 level after the Bonferroni correction is applied

## Conclusions

Previous studies have observed that the odds of quit success for smokers are greater in those who use NRT for a longer duration [8-12]. However, this does not necessarily mean that sending more free NRT to smokers who call a quitline automatically increase their chances of quitting and remaining smokefree. It is possible that the generally higher quit success observed among those reporting a longer duration of NRT is the result of self-selection where those who quit and remain smokefree continue to use their medication while those who relapse discontinue use. For telephone quitlines and other stop programs the question of how much nicotine medication to give smokers to optimize quitting is a very practical one since the desire to promote higher quit rates needs to be balanced against limited resources available to assist clients. This study utilized data collected from different groups of smokers who had contacted the New York Smokers Quiltine over the past 4 years and received different amounts of free nicotine patches to try to answer the question of whether the amount of free patches given to smokers affected quitting outcomes.

The findings from this study do not support the hypothesis that sending more free nicotine patches to smokers' who call a quitline will reliably increase quit rates. 12 month quit rates for each of the five groups were examined in a multivariate logistic regression model adjusting for age, gender, race, education level, and number of cigarettes smoked per day at the time of enrollment. Using the 2-week patch group as the referent category, we found odds ratios of 0.77 (95% CI: 0.49-1.21) for the 4-week patch group; 0.66 (95% CI: 0.40-1.10) for the group who received 6 weeks contingent upon the first 2 weeks of use; 1.55 (95% CI: 1.08-2.21) for the 6-week patch group; and 1.33 (95% CI: 0.89-1.97) for the 8-week patch group. There was not a clear cut dose response relationship observed between the number of free nicotine patches sent to smokers calling quitline and quit rates measured a year later suggesting that it may not be cost-effective to send more than a starter kit of free medications to smokers who call requesting quitting assistance. However, NRT usage patterns may help explain the equivocal cessation outcomes observed. We did examine the correlation between the number of patches used and quitting outcomes in a multivariate logistic regression model adjusting for the same covariates described above. In this model, we did observe a dose response relationship between the number of patches used and odds ratios for quitting. The higher odds ratios for quitting among participants who used a greater number of patches is not unexpected and most likely is the result of self selection where those who quit and remain quit continue to use the medication while those who relapse discontinue their use of medications.

The central goal of our analysis was not to test whether using more patches was associated with higher quit rates, but rather whether the number of free patches sent to smokers calling a quitline would influence quit rates. The answer to this later question seems to be that it does not make a huge difference in quit rates. While those who were sent more free patches reported using more patches, the number of days the patch was used did not differ dramatically between the groups. In other words those who got more free patches appeared to have more left unused compared to those who got only two weeks of free patches. Since the main reason for discontinuing use of the patch is a return to smoking, and since most relapses typically occurs within the first few weeks of quitting, handing out a large supply of free patches at the start of someone's quit attempt does not guarantee a higher quit rate.

Caution is warranted in interpreting results from this study as differences in the characteristics of the groups compared could potentially account for the null findings. A key difference among the five groups of smokers compared in this study was how they were recruited into the different arms. Groups 1, 2 and 3 were largely self-selected callers responding to routine promotions of the NYSSQL. Group 4 participants were all residents of New York City who responded to a time limited, highly publicized offer of free nicotine patches which coincided with the implementation of the City's smoke-free work place law and increase in cigarette taxes. Group 5 participants were unique in that they had all previously called the NYSSQL for quitting assistance and were recruited through a special direct mail campaign at around the same time New York State implemented its comprehensive smoke-free air law. Notably, however, for the two groups whose recruitment was most similar (Groups 1 and 2), there were no differences in outcomes for 2- vs. 4-week supplies of NRT, and none of the other groups had significantly higher quit rates than Group 1 (2-week supply).

Another systematic difference between the groups compared was their medical insurance status. Groups 1 and 2 included only privately insured persons while group 5 included only uninsured persons. Group 3 included Medicaid and uninsured smokers, while group 4 included mainly privately insured persons, but also a mix of publicly insured and uninsured smokers. While previous studies have found an association between medical insurance status and smoking cessation, this association appears to be mediated by motivation to quit smoking and amount smoked daily, both of which are factors that we attempted to control for in this study [20,21]. All five groups were matched on motivation to quit since as a condition of eligibility to get the free patches smokers were required to set a quit date within two weeks of calling the quitline in order to get the free nicotine patches. The groups were also crudely matched on amount smoked per day since as one of the core criteria for eligibility for getting the free patches smokers had to report smoking 10 or more cigarettes per day. Adjustment for amount smoked daily using analysis of covariance also did not change the results.

Another limitation of the study was low follow-up response rates achieved, especially for the Medicaid and uninsured group (group 3), whose overall response rate was only 23 percent and much lower compared to the other four groups. However, our analysis which counted all non-responders as smokers did not alter the overall conclusions reached about group differences.

In the current study, three-quarters or more of the smokers in each of the five study groups reported smoking at follow-up, which, while not unexpected, reflects the obvious point that treatment outcomes still can be improved upon. While the debate about how best to tailor the dose of NRT to smokers remains a hot topic of research in the field of nicotine dependence treatment, few have questioned the value of keeping smokers on NRT for at least 8 to12 weeks [15-17,19,22]. While the optimum duration of treatment needs to be better understood, as important may be the proper dose of medication given to smokers who are trying to quit. Research has previously shown large individual variation in smoking habits and in how different people metabolize nicotine which in turn can influence the how people respond to NRT [23-25]. Some researchers have speculated that treatment outcomes with NRT could be improved if the dose given to smokers trying to quit could be more effectively matched to how they metabolize nicotine [26-28]. In other words, single fixed dose strength of nicotine patches does not necessarily fit all smokers.

It is also possible that quit rates could be improved by adding extra counseling calls beyond the one callback provided to smokers. However, the extra counseling calls provided to the Medicaid and uninsured smoker group in this study did not appear to dramatically increase quit rates over the other groups of smokers that only received one counseling call. This result is consistent with the null results of a recent trial evaluating the benefits of telephone counseling as an adjunct to the use of medications for smoking cessation [29]. However, prior research has also indicated lower cessation rates among Medicaid smokers, so it is possible that the counseling calls improved quit rates over what would have been achieved without the calls extra support calls [20,21]. In addition, it is possible that making receipt of the full 6-week supply contingent on using the first 2-weeks of NRT and completing a proactive call may have influenced both follow-up completion rates and outcomes for this group. Further examination of NRT dosing specifically for Medicaid and uninsured smokers is needed.

Smokers often cite the high cost of NRT as a barrier preventing them from making a quit attempt [19]. Previous studies have demonstrated that offering free NRT can be a safe and cost-effective inducement to get more smokers to call a quitline [1-4]. However, this study reveals that sending more free nicotine patches to smokers who call a quitline does not automatically translate into an enhanced duration of use or higher long term quit rates. Given the inherent limitations in this quasi-experimental study and the largely unchallenged assumption about the importance of longer duration of NRT therapy, we believe a more definitive randomized controlled trial is warranted in order to test the cost-effectiveness of giving smokers different amounts of free NRT when they attempt to quit.

## Competing interests

The authors declare that they have no competing interests.

## Authors' contributions

All authors have read and approved the final manuscript.

Conception and design: KMC, AH, MM, DJO, UB. Acquisition of data: PC. Data analysis and interpretation: KMC, BVF, AH. Drafting the article and/or revising it critically for important intellectual content: KMC, BVF, AH, MM, DJO, UB.

## Pre-publication history

The pre-publication history for this paper can be accessed here:

http://www.biomedcentral.com/1471-2458/10/181/prepub
